# Prenatal diagnosis of accessory mitral valve tissue in a fetus with persistent dysrhythmia

**DOI:** 10.1186/s43044-022-00263-z

**Published:** 2022-04-11

**Authors:** Mohammad Nasir Hematian, Shirin Torabi, Sedigheh Hantoushzadeh, Alireza Dehestani, Minoo Dadkhah, Reza Shabanian

**Affiliations:** 1 Department of Pediatric Cardiology, Yas Hospital Complex, Tehran University of Medical Sciences, Tehran, Iran; 2 Department of Obstetrics and Gynecology, Vali-Asr Hospital, Tehran University of Medical Sciences, Tehran, Iran; 3 Valiasr Maternal, Fetal and Neonatal Research Center, Tehran University of Medical Sciences, Tehran, Iran; 4 Department of Pediatric Cardiac Surgery, Children’s Medical Center, Tehran University of Medical Sciences, Tehran, Iran; 5 Department of Pediatric Cardiology, Baharloo Hospital, Tehran University of Medical Sciences, Tehran, Iran; 6 Department of Pediatric Cardiology, Children’s Medical Center, Tehran University of Medical Sciences, 63 Gharib Street, Tehran, 1419733151 Iran

**Keywords:** Accessory mitral valve tissue, Diverticulum, Fetal echocardiography, Dysrhythmia, Extrasystole

## Abstract

**Background:**

Accessory mitral valve tissue (AMVT) is a rare congenital cardiac anomaly that mainly diagnosed in the first decade of life. However, asymptomatic cases may not be diagnosed even up to adulthood. We report a fetus with AMVT to show the diagnostic ability of the fetal echocardiography for detection of this pathology in the prenatal period.

**Case presentation:**

AMVT was diagnosed in a 26-week-old male fetus with persistent dysrhythmia. Dysrhythmia could not be aborted and controlled by sotalol till the third trimester evaluation. Apical left ventricular (LV) diverticulum was the additional finding in his fetal echocardiogram. After birth, he was in sinus rhythm and echocardiography confirmed the presence of AMVT, however, without any evidence of LV apical diverticulum.

**Conclusions:**

The diagnosis of AMVT in the prenatal period is possible by fetal echocardiography.

**Supplementary Information:**

The online version contains supplementary material available at 10.1186/s43044-022-00263-z.

## Background

Accessory mitral valve tissue (AMVT) is a rare congenital cardiac anomaly. This anomaly is mainly diagnosed in the first decade of life, but it may remain undiagnosed until adulthood [1]. A large proportion of patients with AMVT are asymptomatic and diagnosed incidentally. However, symptomatic patients present with chest pain, dyspnea or arrhythmias. The most serious concern in AMVT is left ventricular outflow tract (LVOT) obstruction that usually requires surgical intervention [2]. To the best of our knowledge, prenatal diagnosis of AMVT has not been previously described. Here, we present a case of non-obstructive AMVT in a fetus of the second trimester that was referred for the assessment of dysrhythmia.

## Case presentation

**Fig. 1:**
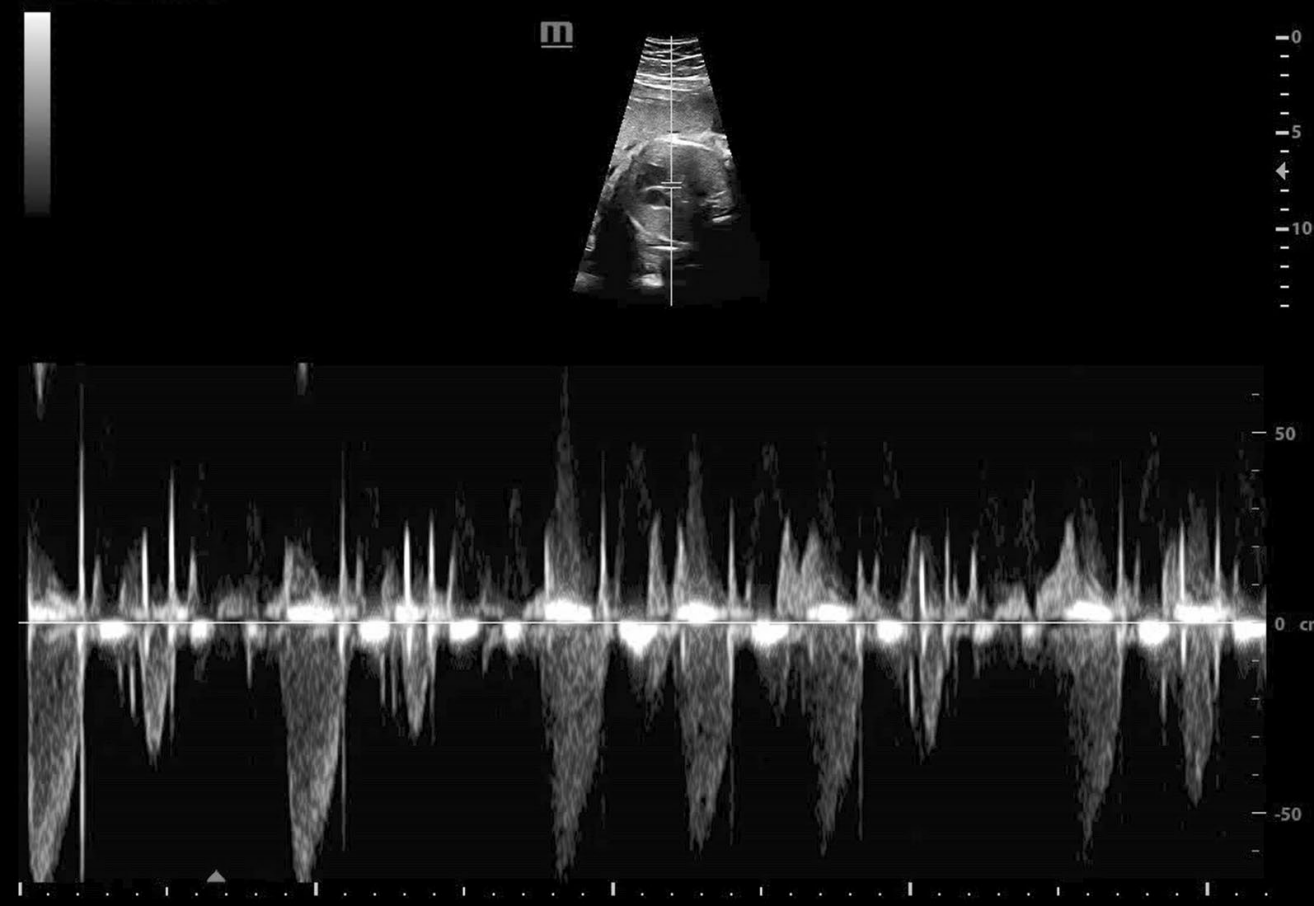
The pulsed Doppler sample gate was placed to include the outflow and inflow of the left ventricle. It shows the occurrence of frequent premature ventricular contractions (PVCs) with the full compensatory pauses and the first part being in a bigeminy pattern

**Fig. 2:**
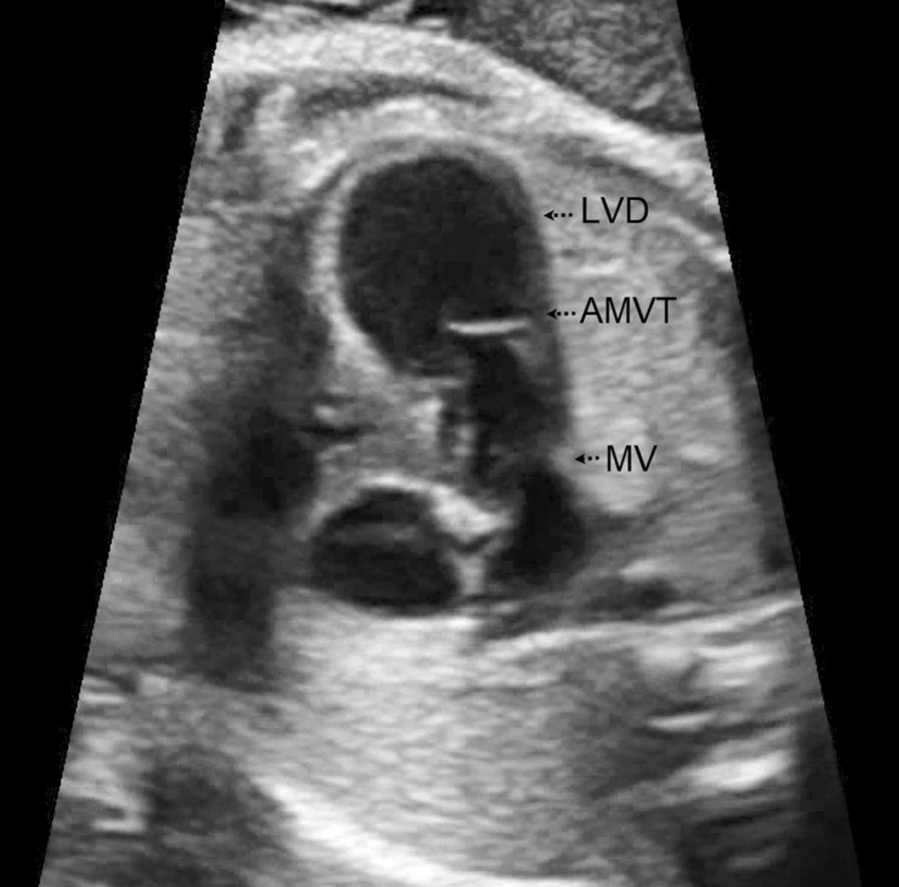
Four chamber view of the heart showing the accessory mitral valve tissue (AMVT) and the outpouching of left ventricular diverticulum (LVD) at 26 weeks of gestation

A 30-year-old pregnant woman (gravid 2, abortion 1 and non-consanguineous marriage) was referred to us at 26 weeks of gestation because of her fetus dysrhythmia. Extrasystoles of bigeminy premature ventricular contractions (PVCs) were the cause of dysrhythmia (Fig. 1). In addition, a contractile outpouching of 7 mm × 10 mm was found in the left ventricular apex (Fig. 2). It was diagnosed as a diverticulum due to its synchronized contraction with the main left ventricular chamber. The diverticulum was connected with a broad neck to the left ventricle and had a low-velocity color flow in it (Additional file 1: Video S1). Given the persistent fetal dysrhythmia, sotalol 80 mg twice a day was initiated for the mother. In the fetal screening examination, no additional extracardiac anomaly was reported. No evidence of cardiac failure or hydrops was noted either. At 32 weeks of gestation, follow-up echocardiography revealed persistent dysrhythmia as PVCs in more than 50% of the time of the test. The LV systolic function and ventricular myocardial performance index were within the normal limits. Hence, it was decided not to continue sotalol. Moreover, an AMVT was identified below the main mitral valve with two leaflets connected to the same papillary muscles by separate chordae tendineae. Leaflets movement and E/A ratio in pulse wave Doppler were similar to the main mitral valve Doppler tracing. Accessory and main mitral valve diameter at the age of 35 weeks of gestation were 6.8 mm and 6.7 mm, respectively. Both valves had simultaneous phases of movement. There was no evidence of valvar regurgitation and LVOT obstruction. The mother's past medical history was unremarkable. She did not report the consumption of any teratogenic drugs or being affected by any viral infections during gestation. Fetal growth scans were normal in the course of pregnancy, and hydrops did not occur either. A baby girl with an Apgar score of 9 and birth weight of 3.1 kg was born at the age of 39 weeks by normal vaginal delivery. She was transferred to NICU for further work-up and monitoring. The electrocardiographic (ECG) tracing after birth was in normal sinus rhythm without any evidence of dysrhythmia. Post-delivery echocardiography confirmed the presence of accessory mitral valve, while the LV apical diverticulum faded away. At 4-month follow-up the infant was free of arrhythmia and LVOT obstruction. She was treated with aspirin, and periodic follow-up was recommended.

## Discussion

Accessory mitral valve tissue as a rare pathology has been reported to have an estimated incidence of 1 out of 26,000 adults [1]. With reporting our case as the prenatal diagnosis of this anomaly, one might claim that AMVT could be an echocardiographic finding from fetal to late adulthood period. Despite the miniaturized anatomy of the fetal heart, the quality and resolution of the fetal echocardiographic images have been remarkably improved since the introduction of this imaging modality [3]. With ongoing evolution in technology, the diagnosis of delicate structural heart defects such as AMVT in the fetus has become possible. Both three-dimensional (3D) and four-dimensional (4D) echocardiography enable to depict a more precise anatomical structure and provide en face visualization of the mitral valve. However, 3D and 4D fetal cardiac imaging require technical expertise [4, 5]. Likewise, although fetal cardiac magnetic resonance imaging has been used as a research tool, its clinical application in fetal heart diagnostic imaging has several limitations [3, 6].

The association of AMVT with other congenital cardiac defects has been frequently reported in the literature [1, 2, 7, 8]. This association might be attributed to the developmental pathophysiology of AMVT in the embryonic period. Normally, the endocardial cushion forms the mitral valve. If this embryonic process fails to develop normally, then AMVT might be separated from the cushion [9]. Major associated anomalies such as ventricular and atrial septal defects, atrial septal aneurysm and subaortic stenosis also originate from the defects in the endocardial cushion development [8].

Several classifications have been used to describe the shape of AMVT in different case series [8–11]. The shape is dependent on the site where AMVT is attached. The site of attachment is on the left-sided heart structures including the anterior and posterior leaflets of the mitral valve, aortic valve, papillary muscles, chordae tendineae, interventricular septum and the left atrial/ventricular walls. For a more precise anatomical description of the lesion, Prifti et al. proposed a classification system [10]. In this system, the accessory valves are divided into two shapes of fixed and mobile types. Fixed types (type I) include nodular (IA) and membranous (IB) accessory valves. Mobile type (type II) is also divided into two groups of pedunculated (IIA) and leaflet likes (IIB) accessory valves. Type IIB is further subdivided as rudimentary or well-developed chorda tendineae. Our case seems to be classified in the subdivision of IIB2 because of its mobile leaflets and well-developed chordae. A simpler classification method by Yetkin et al. [11] uses the true mitral valve leaflets as the primary landmarks for the AMVT typing. In this classification, the three types of I, II and III are described according to the attachments above, on or below the true mitral valve leaflets, respectively.

One-third of the patients with AMVT are asymptomatic. Symptoms of palpitation, chest pain, syncope and dyspnea have been reported. Patients with LVOT obstruction might experience dizziness and exertional dyspnea [8]. Other serious, however, uncommon complications are hemiplegia, transient ischemic attack and retinal artery occlusion due to thromboembolic events [11–13]. Dysrhythmia including atrial fibrillation and ventricular tachycardia has been reported too [8, 11]. The prenatal presentation of ventricular extrasystoles in our fetus might be attributed to the AMVT. Anomalies of the conduction system have been reported in cases of AMVT [14]. However, the co-existence of extrasystoles and AMVT in our case seems to be irrelevant because of the disappearance of both premature beats and LV apical diverticulum in post-partum echocardiograms [15, 16]. The ventricular ectopy and arrhythmia have been reported with LV wall defects including LV diverticulum. Moreover, there are reports of spontaneous fading of ventricular diverticulum before and after delivery [17]. Concomitant disappearance of extrasystoles and diverticulum in our patient's follow-up might be a clue for their cause and effect relationship.

Some studies have reported that AMVTs might be overlooked in the echocardiographic examination for other cardiac anomalies, and even some cases of AMVT have been detected years after cardiac surgery. Hence, it is advised to improve echocardiographic imaging especially in the field of fetal echocardiography by optimizing image quality in different aspects of probe resolution, probe position, transducer pressure, depth and sector width.

Clinical assessment of patients is necessary for choosing the treatment in patients with AMVT. In asymptomatic patients without any associated conditions, only follow-up is recommended. However, in symptomatic patients, multiple factors including the symptoms, obstruction of the LVOT, valvar disease, heart failure and the concomitant anomalies must be considered in the therapeutic approach [8]. Antiplatelet therapy has been suggested in all patients with AMVT [13]. Surgery is a choice with relatively vague indications in the literature. Overall, surgical resection is indicated in symptomatic patients or those with prominent LVOT obstruction [1, 8].

## Conclusions

The AMVT is a rare cardiac anomaly which could be diagnosed in prenatal period in association with other structural cardiac anomalies such as ventricular diverticulum or presented as fetal dysrhythmia including ventricular premature beats.

## Supplementary Information

**Additional file 1: Video S1.** 2D and color Doppler four chamber view of the heart showing type IIB2 accessory mitral valve tissue (AMVT) and the synchronous left ventricular diverticulum (LVD) contraction with the main LV chamber at 26 weeks of gestation.

## Data Availability

Please contact the corresponding author for data requests.

## Abbreviations

AMVT: Accessory mitral valve tissue
ECG: Electrocardiography
LV: Left ventricle
LVOT: Left ventricular outflow tract
PVC: Premature ventricular contraction
